# A Prevalent Focused Human Antibody Response to the Influenza Virus Hemagglutinin Head Interface

**DOI:** 10.1128/mBio.01144-21

**Published:** 2021-06-01

**Authors:** Kevin R. McCarthy, Jiwon Lee, Akiko Watanabe, Masayuki Kuraoka, Lindsey R. Robinson-McCarthy, George Georgiou, Garnett Kelsoe, Stephen C. Harrison

**Affiliations:** a Laboratory of Molecular Medicine, Boston Children’s Hospital, Boston, Massachusetts, USA; b Harvard Medical School, Boston, Massachusetts, USA; c Thayer School of Engineering, Dartmouth College, Hannover, New Hampshire, USA; d Department of Immunology, Duke University, Durham, North Carolina, USA; e Department of Chemical Engineering, University of Texas at Austin, Austin, Texas, USA; f Department of Molecular Biosciences, University of Texas at Austin, Austin, Texas, USA; g Department of Biomedical Engineering, University of Texas at Austin, Austin, Texas, USA; h Department of Oncology, University of Texas at Austin, Austin, Texas, USA; i Howard Hughes Medical Institute, Boston, Massachusetts, USA; Washington University School of Medicine

**Keywords:** X-ray crystallography, conserved epitope, human antibody repertoire, influenza vaccines

## Abstract

Novel animal influenza viruses emerge, initiate pandemics, and become endemic seasonal variants that have evolved to escape from prevalent herd immunity. These processes often outpace vaccine-elicited protection. Focusing immune responses on conserved epitopes may impart durable immunity. We describe a focused, protective antibody response, abundant in memory and serum repertoires, to a conserved region at the influenza virus hemagglutinin (HA) head interface. Structures of 11 examples, 8 reported here, from seven human donors demonstrate the convergence of responses on a single epitope. The 11 are genetically diverse, with one class having a common, IGκV1-39, light chain. All of the antibodies bind HAs from multiple serotypes. The lack of apparent genetic restriction and potential for elicitation by more than one serotype may explain their abundance. We define the head interface as a major target of broadly protective antibodies with the potential to influence the outcomes of influenza virus infection.

## INTRODUCTION

Influenza viruses threaten human health as both endemic (seasonal flu) and emerging (pandemic flu) pathogens. Four distinct seasonal influenza virus subtypes currently circulate each flu season. Each subtype evolves to escape dominant protective herd immunity elicited by past influenza exposures, a process known as antigenic drift. Antigenically diverse influenza viruses emerge from animal reservoirs to initiate pandemics that sweep through immune-naive human populations; between 1918 and 2009, this “antigenic shift” has occurred at least four times. Improved influenza vaccines will need to impart both seasonal and prepandemic immunity.

Focusing antibody responses on conserved epitopes is at the foundation of efforts to make improved or universal flu vaccine immunogens. Known broadly protective antibodies engage sites on the major virion surface protein, influenza virus hemagglutinin (HA). These include the receptor-binding site (RBS) on the “head” of an HA subunit (1–5), sites on the HA “stem” (6, 7), and a recently identified site at the interface between two heads of an HA trimer (4, 8–10). Antibodies to the RBS are potently neutralizing. The importance of residues at the periphery of the RBS for defining antigenic clusters suggests, however, that the most common of the RBS-directed antibodies are sensitive to the identity of those residues, probably limiting the long-term breadth of any such antibodies with footprints that extend beyond the sialic acid pocket (11). Stem and head interface antibodies realize their full protective potential through Fc-dependent pathways that lead to the killing of infected cells rather than by blocking viral entry (12, 13). Broadly protective antibodies directed at these epitopes are likely present in most flu-exposed humans (3, 5, 9, 14–16).

We have found that seasonal flu vaccination elicits particularly strong serum antibody and memory B-cell responses directed at the HA head interface. We describe here the anatomy of this dominant and focused response by examining HA-Fab structures of 11 antibodies (including 8 reported here) isolated from seven human subjects. The head interface accommodates contacts from structurally and, correspondingly, genetically diverse antibodies. A distinct subset (five antibodies from five different subjects) has a light chain variable region encoded by the IGκV1-39 gene. Members of this germ line-restricted subset bind HA nearly identically, and we define from structures the molecular basis for this restriction. All of the characterized antibodies bind to multiple HA serotypes, suggesting that the epitope may elicit an intrinsically broad response. These qualities of the head interface epitope can inform the design of improved influenza vaccines.

## RESULTS

### Origins of antibodies in this study.

The structures of three HA-bound head interface antibodies (H2214, S5V2-29, and FluA-20), from three different donors (designated EI-13, S5, and FluA-20 donor), have been reported previously (9, 10, 17). We have now characterized six additional head interface antibodies from two further donors (S1 and S8), identified by antibody/Fab competition. Antibodies D1 H1-3/H3-3, D1 H1-17/H3-14, and D2 H1-1/H3-1 were isolated from donors designated D1 and D2, respectively, and described originally as engaging the opposite surface of the contact between heads (4). We determined that these antibodies instead compete for binding with an H2214 Fab on monomeric HA heads (see Fig. S1A in the supplemental material). Lineage analysis (18) suggests that both antibodies from D1 have arisen from a single naive progenitor. We therefore assembled a panel of 11 unrelated antibodies, from seven donors, representing nearly all described examples of head interface antibodies.

**FIG 1:**
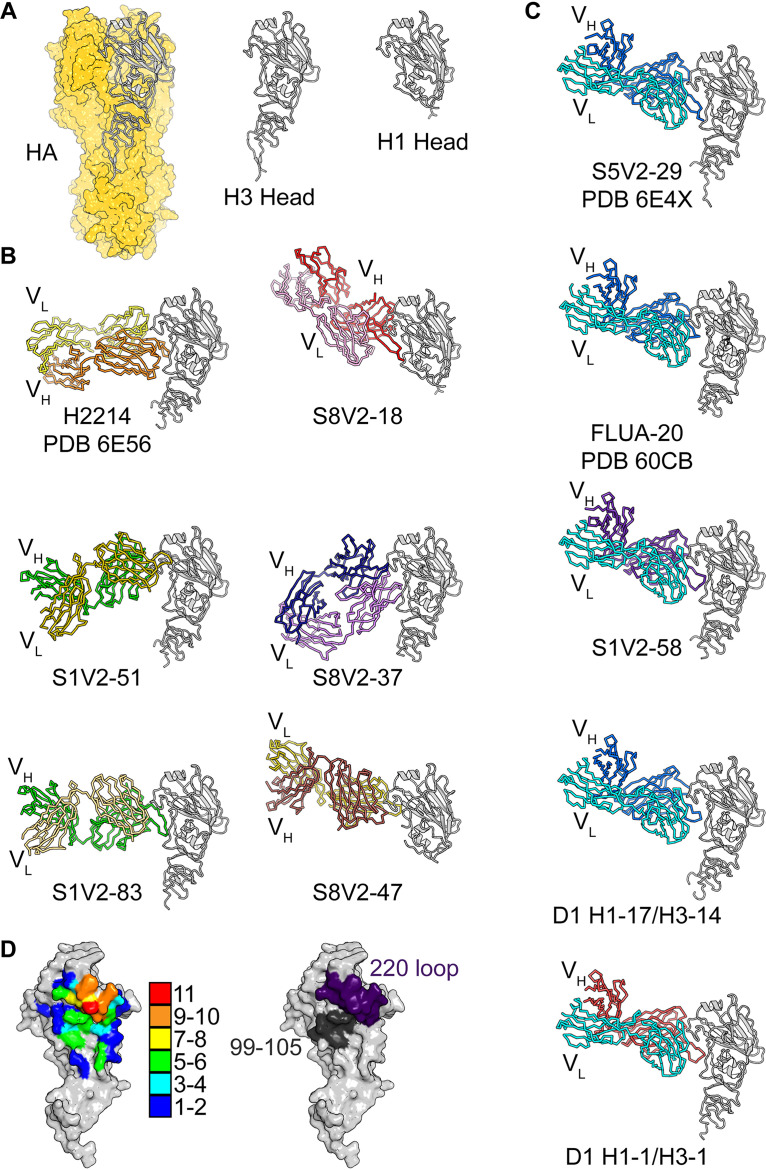
Structures of head interface antibodies bound to HA head domains. (A) HA trimer from A/Bangkok/01/1979(H3N2), shown with a yellow surface. The HA head domain of a single protomer is shown in a cartoon representation in gray. The H3 and H1 head constructs used for crystallization experiments are shown to the right. Fabs are colored according to the V_H_ (darker color) and V_L_ (lighter color) gene usage. HA heads are all shown in gray. H2214, S5V2-29, and FluA-20 have been previously reported, and their PDB accession numbers are indicated (9, 10). (B) The six antibodies (two columns of three) that do not use the IGκV1-39 gene. (C) One column showing the five antibodies that use IGκV1-39 (cyan). (D, left) Contact heat map on the HA head surface. The numbers of Fabs (from a total of 11) contacting each residue were tallied and are colored according to the key. Contacts for IGκV1-39 Fabs and non-IGκV1-39 Fabs and their relationship with conserved residues are shown in Fig. S1C in the supplemental material. (Right) Two key points of contact, the 220 loop (purple) and residues 99 to 105 (dark gray), are shown on the HA surface.

10.1128/mBio.01144-21.1 FIG S1 Head interface antibodies. (A) Identification of additional head interface antibodies. Panels show traces for the association and dissociation of an HA head domain of A/Texas/50/2012(H3N2), at a concentration of 12 μM, with the Fab fragment of the antibody shown in the panel header, immobilized on a BLI sensor. In each panel, the blue curve shows binding in the absence of any competitor, the red curve shows binding in the presence of a 4-fold molar excess of H2214 (head interface directed) (A. Watanabe, K. R. McCarthy, M. Kuraoka, A. G. Schmidt, et al., Cell 177:1124–1135.e16, 2019, https://doi.org/10.1016/j.cell.2019.03.048) preincubated with the HA head, the black curve shows binding in the presence of a 4-fold molar excess of Fab from RBS-directed antibody K03.12 (K. R. McCarthy, A. Watanabe, M. Kuraoka, K. T. Do, et al., Immunity 48:174–184.e9, 2018, https://doi.org/10.1016/j.immuni.2017.12.009) preincubated with the HA head, and the gray curves for D1 and D2 antibodies show competition with the antibody named in the panel header in the presence of a 4-fold molar excess of Fab (competition with itself). (B) Gene usage, H/LCDR3 sequences, and percent somatic hypermutation (SHM) of human HA head interface antibodies discussed in this article. (C) Relationship between Fab-HA contacts and conserved surfaces. HA head domains are shown. The first three show contact heat maps for interface-directed antibodies. Each is colored according to the key below each HA head. From left to right, they are shown as the 11 examples from Fig. 1: the 5 that use the IGκV1-39 light chain and the 6 that do not use this light chain. Conservation across sialic acid-binding influenza A virus serotypes (H1 to H16), group 1 (H1, -2, -5, -6, -8, -9, -11, -12, -13, and -16) and group 2 (H3, -4, -7, -10, -14, and -15) HAs, are colored according to the key below. Download FIG S1, TIF file, 2.7 MB. Copyright © 2021 McCarthy et al. 2021 McCarthy et al. https://creativecommons.org/licenses/by/4.0/ This content is distributed under the terms of the Creative Commons Attribution 4.0 International license.

These antibodies arose from several different combinations of V, D, and J gene segments (Fig. S1B). Combinations of nine V_H_, all from the IGHV-3 and IGHV-4 gene families; six D_H_; and four J_H_ gene segments encoded the 11 heavy chain variable domains. HCDR3 lengths ranged from 9 to 25 amino acid residues. Six different κ_V_ and four κ_J_ gene segments (among which 2*01 appeared five times) encoded the 11 light chains, all V-kappa. In 5 of the 11, IGκV1-39 had recombined with four different κ_J_ gene segments. LCDR3 lengths were between 5 and 10 residues.

### Structures.

Structures of HA-bound Fab fragments of S5V2-29, H2214, and FluA-20 have been reported previously (9). We determined eight additional structures of Fab fragments from S1V2-51, S1V2-58, S1V2-83, S8V2-18, S8V2-37, S8V2-47, D1 H1-17/H3-14, and D2 H1-1/H3-1 in complex with various HA head domains (Fig. 1A and B). All engage the same lateral surface of the HA head, although they do so at various angles and in various orientations with reference to the heavy and light chains. Despite the diversity of paratopes, all 11 Fabs ultimately converge upon a common site. The five antibodies with light chains encoded by the IGκV1-39 gene segment, each from a different donor, all engage HA similarly (Fig. 1C).

We have used these 11 structures to define the common head interface epitope. The heat map in Fig. 1D illustrates a focused response, centered on the HA-220 loop. All 11 antibodies contact Pro221, and 10 of the 11 contact residues 222, 223, and 229. Other positions within this loop have contacts from more than half of the antibodies. The second most frequently contacted segment includes HA residues 99 to 105. Less frequently contacted sites ring these two “hot spots.”

### IGκV1-39 germ line bias.

**FIG 2:**
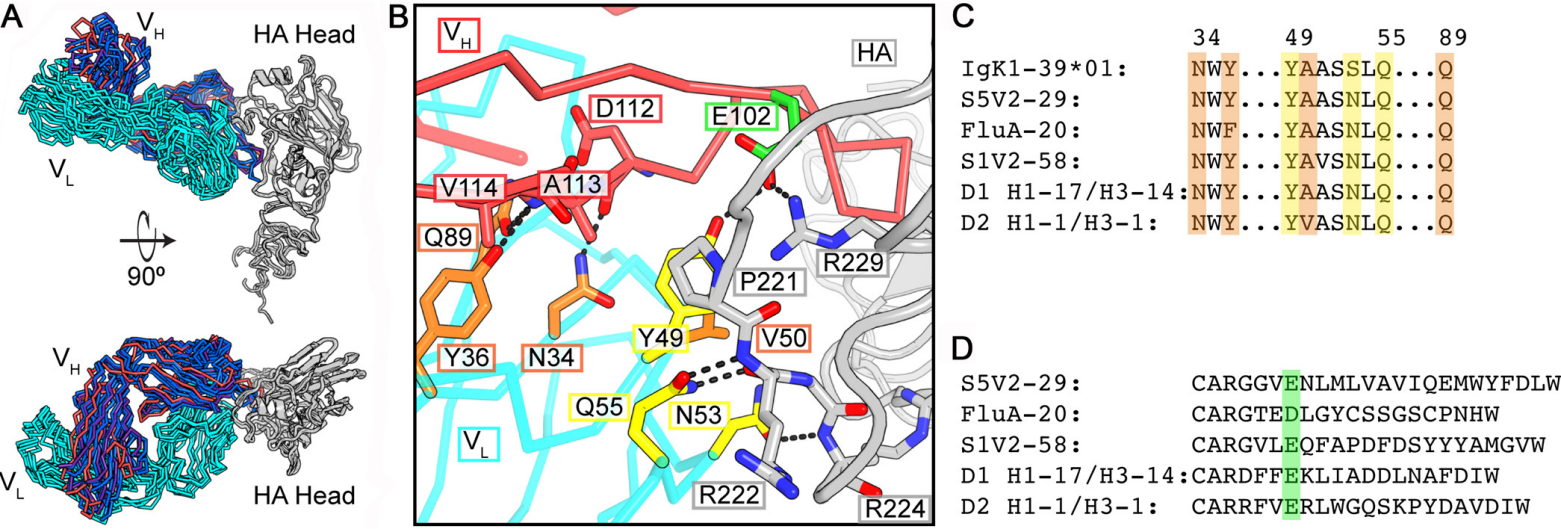
Stereotyped HA engagement by IGκV1-39 antibodies. (A) Superposition on the HA head domain of the five IGκV1-39 antibodies. The HA heads are in gray, IGκV1-39 is in cyan, and heavy chains are colored according to V_H_ gene usage and are the same as those in Fig. 1. (B) Zoomed-in view of the IGκV1-39 interaction of D2 H1-1/H3-1 with HA from the bottom view of panel A. Key residues are shown in sticks. Light chain residues that contact HA are in yellow, and those that buttress/permit a curled HCDR3 conformation are in orange. A common acidic residue in HCDR3 is highlighted in green. (C) Partial sequence alignment of the germ line IGκV1-39 sequence and the five antibodies. Shading is colored as described above for panel B. (D) Sequence alignment of the antibody HCDR3s. The acidic residue at the sixth position of HCDR3 is shaded in green.

Among the five IGκV1-39-encoded antibodies, four have heavy chains encoded by genes recombined from IGHV4-61 or IGHV4-59, and one, D2 H1-1/H3-1, has the heavy chain from IGHV3-7. These antibodies present HA with a similar paratope. The HCDR3s, while unique in sequence and length, all curl toward the light chain and create a blunt antigen-combining site that brings light chain framework regions 2 and 3 into contact with HA (Fig. 2 and Fig. S1B). This arrangement accommodates the HA-220 loop in a groove at the heavy-light chain interface, with HCDR3 tucked beneath it.

From these structures and the sequences of the human light chain V-gene repertoire, we can infer an IGκV1-39 signature to explain the stereotyped HA interaction. The principal contacts between the light chain and HA are from residues 49 to 55: YAASSLQ in the germ line sequence (Fig. 2B and Fig. S2). In all five structures, the side chain of Gln at position 55 hydrogen bonds with both the main-chain NH and the main-chain CO of HA residue 222. Also, in all five structures, a Ser53Asn substitution allows the Asn side chain to accept a hydrogen bond from the main-chain NH of HA residue 224 while donating a branched hydrogen bond to the carbonyls of residues 49 and 50 in the β-turn at the tip of the LCDR2 loop. The side chain of Tyr49, anchored by a hydrogen bond with an acidic residue at the sixth position in HCDR3 in all five antibodies (Fig. 2), is in van der Waals contact with conserved HA Pro221. The HCDR3 acidic residue (supplied by IGHD3-3*01 or by n-nucleotides) then forms a double salt bridge with conserved HA-Arg229. The Tyr49-HCDR3-Asp/Glu interaction and Tyr49-HCDR3 van der Waals interactions buttress the curl of HCDR3. The small amino acid residue at light chain position 50 avoids clashes with HCDR3 and HA while making van der Waals contacts with each. Additional HCDR3 stabilization appears to come from main-chain hydrogen bonds with side chains of light chain residues Asn34, Tyr36 (Phe in FluA-20, perhaps dispensable after affinity maturation), and Gln89. The motif 49-Y-small-X-X-N-X-Q-55, Asn34, Tyr36, Gln89 is unique to IGκV1-39 (Fig. 2B to D and Fig. S2). Because much of this motif is germ line encoded, it also likely explains the abundance of this gene segment among those encoding the light chains of these antibodies.

10.1128/mBio.01144-21.2 FIG S2 Identification of the genetic contribution for the IGκV1-39 gene bias. (A) Workflow using a motif search informed by structural analysis. (B) Alignment of V genes that fulfill the sequence motif search. Residues in the motif are in red. Residues that buttress HCDR3 are denoted with orange triangles. Only alleles or an identical duplication of IGκV1-39 has both the motif and buttressing residues. (C) Sequence alignment of the germ line IGκV1-39 and the IGκV1-39 antibodies that were characterized. Key residues are shown in red. (D) Codon analysis of the S53N mutation that is present in all IGκV1-39 antibodies. Codons 52, 53, and 54 are shown. The S53N mutation is due to a single nucleotide change (red), introduced by AID. The cytosine occurs in an AID hot spot sequence motif. Download FIG S2, TIF file, 2.8 MB. Copyright © 2021 McCarthy et al. 2021 McCarthy et al. https://creativecommons.org/licenses/by/4.0/ This content is distributed under the terms of the Creative Commons Attribution 4.0 International license.

### Unique examples.

**FIG 3:**
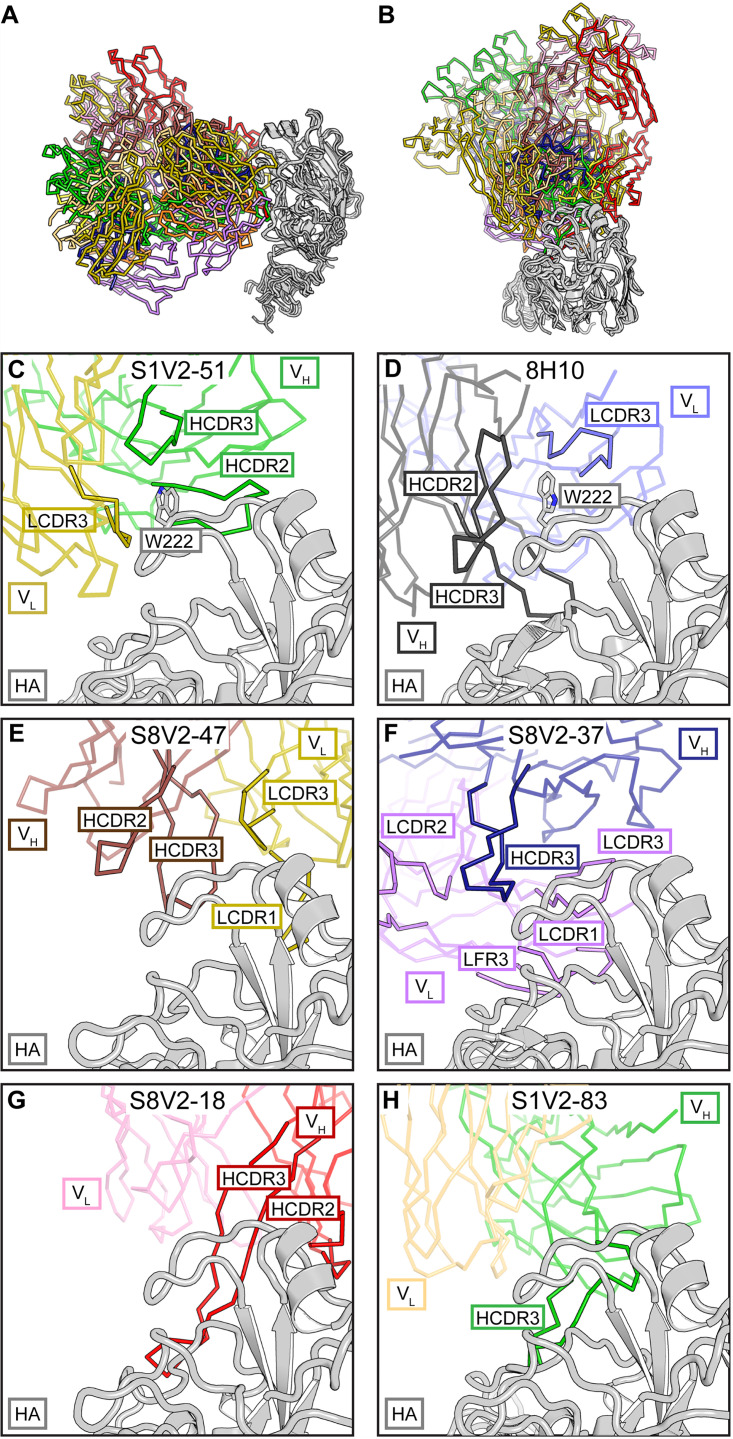
Diversity of non-IGκV1-39 head interface antibodies. (A) All six structures are shown with a common orientation for the HA head domain. Fabs are colored according to V_H_ (darker color) and V_L_ (lighter color) gene usage; HA heads are in gray. Some V-gene usages are shared among these antibodies, but they do not dictate how those Fabs contact HA. (B) Top views of the structures in panel A. Panels C to H are details of this view. Fab fragments are faded except for selected complementarity determining regions (CDR) or framework regions (FR) to highlight the diversity of Fab-HA contacts. (C) S1V2-51. HA residue Trp222 is coordinated in a hydrophobic vault by HCDR2 and -3 and LCDR3 that is similar to mouse antibody 8H10. (D) 8H10 (8) (PDB accession number 6N5B) and its interaction with Trp222. (E) S8V2-47. HCDR2 and -3 and LCDR1 and -3 all contribute to the interaction with HA. (F) S8V2-37. HCDR3 occupies the position of LCDR2 in the IGVκ1-39 antibody complexes and creates an intertwined antigen-combining site with HCDR3, LCDR1 and -2, and LFR3. (G) S8V2-18. Among the interface antibodies, HCDR2 and -3 contact the most recessed surfaces of the HA head. (H) S1V2-83. Most of the interaction with HA is through HCDR3.

The six antibodies with other light chains are quite diverse in the ways in which they contact HA (Fig. 3A and B). For these antibodies, gene usage does not correlate with HA contacts. As an ensemble, they represent the products of multiple alternative affinity maturation pathways for head interface engagement. We describe below the five newly reported here.

S1V2-51 has a short, 9-amino-acid HCDR3 that lies within the heavy-light chain interface and creates a hydrophobic vault into which HA Pro221 and Trp222 insert (Fig. 3C). HCDR3 caps the vault and supplies polar residues, including Asp99, which forms a salt bridge to the Nε1 of Trp222. HA engagement is similar to those of murine interface antibodies that also coordinate Pro221 and Trp222 within the hydrophobic heavy-light chain interface (Fig. 3D). The human and mouse antibodies also contact residues of the receptor-binding site. In S1V2-51, a Phe extends from a long CDRL1 to make van der Walls contacts with the conserved sialic acid-coordinating residue Leu226. S8V2-47 uses the same light chain V segment as S1V2-51, IGκV4-1*01. In these examples, HA binding is not biased by gene usage as their light chains engage opposite sides of the head interface epitope (Fig. 3C and E). In S8V2-47 the HA-220 loop, including Pro221, is pinched between HCDR2 and -3 (Fig. 3E).

The approach of S8V2-37 is substantially different from those of the other interface antibodies, as it engages HA with a distinctive antigen-combining site. HCDR3 has displaced LCDR2 and flanking parts of framework regions 2 and 3 from their positions in the IGVκ1-39 antibody complexes, creating an intertwined paratope (Fig. 3F). Contacts with HA are nearly all through HCDR3, LCDR1, LCDR3, and light chain framework 3.

S8V2-18 contacts HA almost entirely through its heavy chain due to a long HCDR3. The light chain contributes one van der Walls interaction with HA Pro221. S8V2-18 contacts the most recessed surfaces of any of the interface antibodies. Its heavy chain hooks around the HA head and contacts residues facing the 3-fold axis of the trimer (Fig. 3G). S1V2-83 has the longest HCDR3 (25 amino acids), which adopts a Γ-shaped conformation. The arm contacts conserved sites on the HA-220 loop and its stem extending downward along the interface epitope (Fig. 3H). This interaction contributes most of the contacts with HA. Both S1V2-83 and S1V2-51 are V(D)J recombinants derived from IGH3-30*01; this gene usage does not appear to impose a biased mode of binding (Fig. 3C and H).

### Broad HA reactivity of interface antibodies.

**FIG 4:**
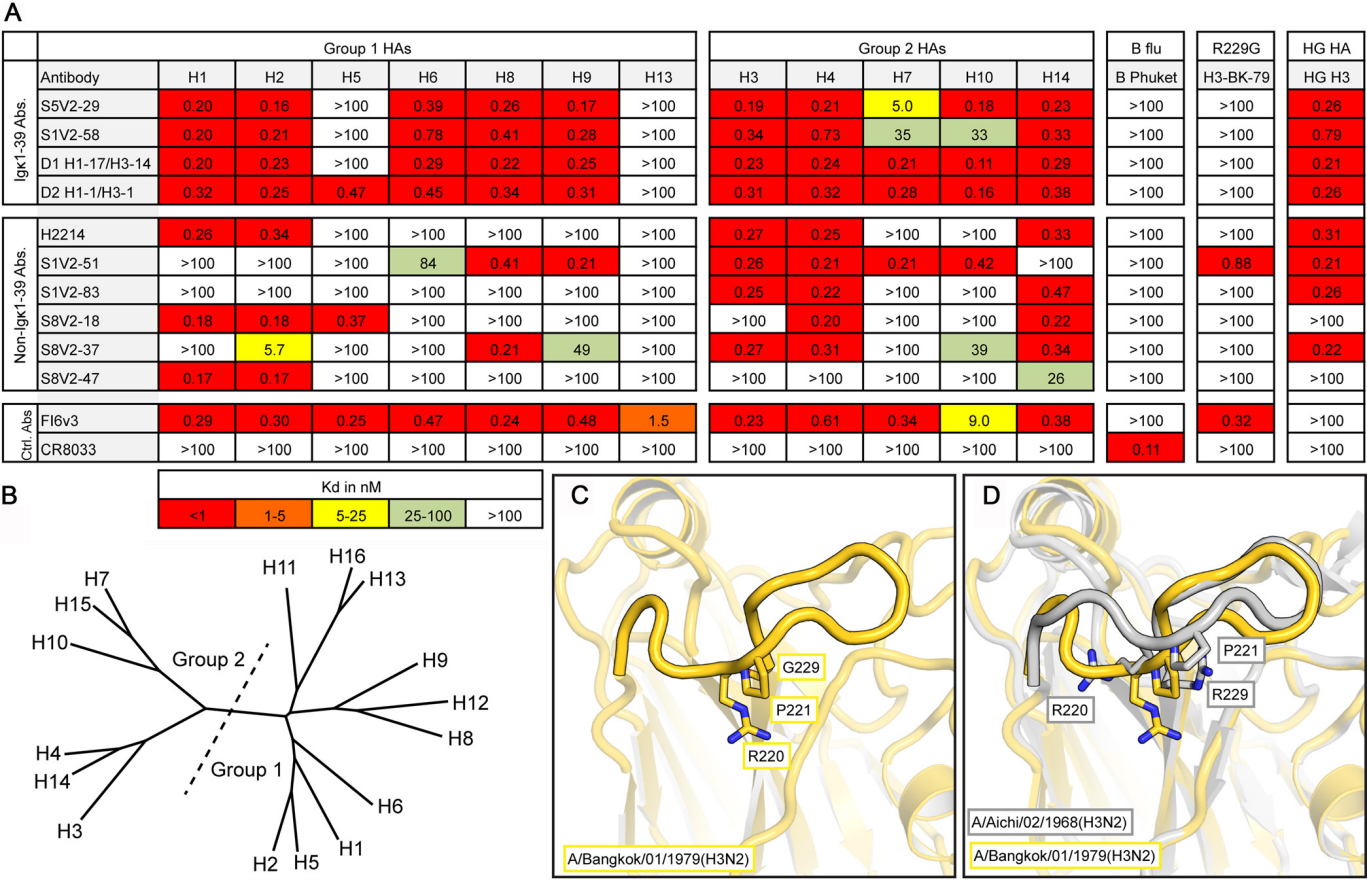
Breadth of binding by head interface antibodies (Abs). (A) Dissociation constants from ELISA measurements. The pan-influenza A virus-binding stem-directed antibody FI6v3 (12) was used as a positive control, and an influenza B virus-specific RBS-directed antibody, CR8033 (31), was used as a negative control for influenza A virus isolates. Boxes are colored according to the key at the bottom. HAs used are as follows: H1 A/California/04/2009(H1N1), H2 A/Japan/305/1957(H2N2), H3 A/Hong Kong/JY2/1968(H3N2), H4 A/American black duck/New Brunswick/00464/2010(H4N6), H5 A/Viet Nam/1203/2004(H5N1), H6 A/Taiwan/02/2013(H6N1), H7 A/Taiwan/01/2017(H7N9), H8 A/northern shoveler/California/HKWF1204/2007(H8N4), H9 A/Beijing/1/2017(H9N2), H10 A/Jiangxi/IPB13/2013(H10N8), H13 A/gull/Maryland/704/1977(H13N6), A/mallard/Wisconsin/10OS3941/2010(H14N6), B-Phuket B/Phuket/3073/2013, H3-BK-79 A/Bangkok/01/1979(H3N2), and gHA^shield^ (8). (B) Phylogram of 16 HA serotypes. The division of HA groups 1 and 2 is shown by a dotted line. (C) Structure of the 220 loop of A/Bangkok/01/1979(H3N2). The HA head is faded except for the 220 loop to emphasize this feature. Residues Arg220, Pro221, and Gly229 are shown in sticks. (D) Comparison with A/Aichi/02/1968(H3N2) (PDB accession number 2VIU) (gray), which has an Arg at position 229.

We examined the binding of these antibodies to divergent HA subtypes by an enzyme-linked immunosorbent assay (ELISA) (Fig. 4A and B). All of them bound HAs from at least two serotypes, and all but one bound at least one group 1 and group 2 HA. The antibodies belonging to the IGκV1-39 subclass had the greatest breadth of binding. Three of these antibodies bound to 10 and one bound to 11 of the 12 HA serotypes assayed. The combined breadth of the subject 8 antibodies, without an IGκV1-39 representative, covered 10 of the 12 HA serotypes, demonstrating that polyclonality and diverse V(D)J gene usage can achieve broad reactivity.

The polyclonality of the head interface response should, in principle, prevent panvirus resistance. Among circulating strains of most HA serotypes, Arg229 is maintained by strong purifying selection. Some laboratory-passaged viruses have a substitution for this residue (9). Most head interface antibodies contact Arg229 (Fig. 1D) and fail to bind 229 mutants (Fig. 4A). We determined the HA trimer structure of laboratory-propagated A/Bangkok/01/1979(H3N2) with its acquired Arg229Gly substitution. The mutation led to a distortion of the 220 loop, the major contact site for all head interface antibodies (Fig. 1D and Fig. 4C and D). The reconfigured loop in our structure was unlike that in other HAs in the PDB (aside from one other R229 mutant, under PDB accession number 6MXU). Despite the substitution and rearrangement, S1V2-51 bound the A/Bangkok/01/1979(H3N2) isolate (Fig. 4A). Constraints on the 220 loop, which forms one side of the receptor-binding pocket, are likely to limit potential pathways of antibody escape.

We also assayed binding to a hyperglycosylated HA immunogen, gHA^shield^ (8), based on an A/Aichi/02/1968(X-31)(H3N2) template (Fig. 4A). Mice immunized with gHA^shield^ have higher frequencies of head interface antibodies than do those immunized with the wild type (WT) (8). All of the human antibodies that bind wild-type A/Aichi/02/1968(X-31)(H3N2) (*n* = 8) also bind gHA^shield^ with comparable affinities. Similar immunogens may boost or selectively elicit the desired classes of head interface antibodies in humans.

## DISCUSSION

Influenza virus HA head interface-directed circulating memory B cells are abundant in humans following flu vaccination, as are interface-directed serum antibodies. In donors S1, S5, and S8, ∼4% of the influenza A virus-reactive memory B-cell repertoire encoded interface-directed B-cell receptors (BCRs) (9). Serum antibodies from donors D1 and D2 were quantified by mass spectrometry (4). In the H1-directed response, antibody D2 H1-1/H3-1 ranked first and accounted for >15% of the influenza A virus antibody repertoire at day 28 postvaccination. The two D1 antibodies (H1-3/H3-3 and H1-17/H3-14) appear to be clonally related; H1-3/H3-3 is the third most abundant antibody in the vaccine responses to both the H1 and H3 components, together accounting for about 11% of the influenza A virus antibody repertoire at day 28. These antibodies were also present prevaccination, reflecting the abundance of cells with the same clonotype in the long-lived plasma cell compartment (4). Thus, the influenza virus HA head interface is immunogenic, and it elicits a strong serum response and robust seeding of B-cell memory. The structures described here define the anatomy of this dominant, focused antibody response to a single epitopic surface.

Contacts with HA from the 11 antibodies (8 newly reported here) from seven human donors overlap each other substantially and define the core epitope. They show that structurally and genetically diverse antibodies can engage this core while varying slightly in their interactions with its periphery. One antibody subclass has a mode of HA binding dictated by the IGκV1-39 gene. The five examples from five human donors bind nearly identically. Apparent determinants for this bias are largely germ line encoded; they are unique to IGκV1-39*01 (or an identical duplicated copy of IGκV1-39*01). Within the IGκV1-39 subclass of interface antibodies, common substitutions reflect convergent affinity maturation pathways, as does the presence of a D-gene- or n-nucleotide-encoded residue in HCDR3. The acquired substitutions, all at the same nucleotide, occur in an activation induced cytidine deaminase (AID) mutation hot spot, perhaps contributing to the abundance of this subclass (see Fig. S2 in the supplemental material).

Why is the interface epitope immunogenic at all, since it is largely occluded in the “ground state” conformation of the trimer? The failure of interface-directed antibodies to neutralize in single-round infectivity assays suggests that, like the HA stem, the epitope is poorly accessible on virions (8–10). Nonetheless, members of both of these classes of nonneutralizing antibodies protect mice from lethal challenge by Fc-mediated mechanisms, implying that the corresponding epitopes are more readily accessible when HA is present on the cell surface. Since all but one of the samples from which the antibodies in this study were obtained were taken following immunization with tetravalent or quadrivalent influenza vaccine (and, hence, exposure to isolated protein rather than intact virions), conformational fluctuations in the split-vaccine HA immunogen would probably have been at least as pronounced as it is for HA on the surface of a cell. Moreover, our incomplete understanding of how and in what form antigen is presented to B cells by follicular dendritic cells in germinal centers leaves open additional mechanisms for the strength of a secondary response to one epitope with respect to another.

Conservation of the head interface may also contribute to its apparent immunodominance. Imprinting by an initial exposure appears to condition all later responses to influenza virus HA. A conserved epitope, even if only transiently exposed, may become immunodominant “by default” if most other immunogenic epitopes have mutated. For example, conservation in most H1 isolates since 1918 of a lateral patch on the head of H1 HAs caused this epitope to dominate humoral responses to the 2009 pandemic H1, which differed on other exposed surfaces from the variants most living individuals would have seen (19, 20). Unlike the head interface, the lateral patch has no apparent structural or functional role, and mutations quickly appeared in circulating HAs, leading to the prevalence of the resistant viruses within 2 to 3 years (19). Thus, even an initially subdominant epitope can become dominant if it is the only one well represented in the memory compartment.

Stem-directed antibodies appear to be less abundant than interface-directed ones despite broad conservation of the relevant surface. As we have shown here, the latter have diverse germ line origins and few paratopic constraints, while the former have constrained gene usage and limited paratopic diversity (14–16). The number of naive, mature B cells in a population expressing randomly recombined BCRs should therefore be much higher for the genetically diverse interface response than for the more restricted stem response. Moreover, the frequency of H1-H3 cross-reactive interface antibodies implies that imprinting by either an H1 or an H3 subtype would establish a common memory pool. For the head interface-directed antibodies, V_H_ mutation frequencies ranged between 3.5 and 10%, consistent with recall responses and comparable to the levels observed for stem-directed antibodies.

A major open question is whether antibodies to the stem or interface afford protection or reduce the severity of infection in humans. Preexisting, but incomplete, immunity to influenza virus can lessen disease severity and duration, as documented for flu seasons in which circulating and vaccine isolates are antigenically mismatched (21–23); effector functions of nonneutralizing antibodies might account for some of this reduction. For conserved surfaces such as the head interface, such antibodies would also confer some degree of prepandemic protection (9, 10). Vaccines that elicit disease-burden-limiting immunity might thus prove valuable for both recurrent seasonal infections and emerging viruses.

## MATERIALS AND METHODS

### Cell lines.

Human 293F cells were maintained at 37°C with 5% CO_2_ in FreeStyle 293 expression medium (Thermo Fisher) supplemented with penicillin and streptomycin. High Five cells (BTI-TN-5B1-4) (Trichoplusia ni) were maintained at 28°C in Ex-Cell 405 medium (Sigma) supplemented with penicillin and streptomycin.

### Recombinant Fab expression and purification.

Synthetic heavy and light chain variable domain genes for Fabs were cloned into a modified pVRC8400 expression vector, as previously described (5, 24, 25). Fab fragments used in crystallization were produced with a C-terminal, noncleavable six-histidine (6×His) tag. Fab fragments used for binding studies were cloned into a pVRC8400 vector that was further modified by the introduction of a rhinovirus 3C protease cleavage site between the heavy chain constant domain and a C-terminal 6×His tag (5). Fab fragments were produced by polyethylenimine (PEI)-facilitated transient transfection of 293F cells that were maintained in FreeStyle 293 expression medium. Transfection complexes were prepared in Opti-MEM and added to cells. Supernatants were harvested at 4 to 5 days posttransfection and clarified by low-speed centrifugation. Fabs were purified by passage over cobalt-nitrilotriacetic acid (Co-NTA) agarose (Clontech) followed by gel filtration chromatography on a Superdex 200 column (GE Healthcare) in a solution containing 10 mM Tris-HCl and 150 mM NaCl at pH 7.5 (buffer A). For binding studies, the 6×His tags were removed from some Fabs by treatment with PreScission protease (MolBioTech, Thermo Scientific), and the protein was repurified on Co-NTA agarose (Clontech) followed by gel filtration chromatography on a Superdex 200 column (GE Healthcare) in buffer A to remove the protease, tag, and uncleaved protein.

### Recombinant IgG expression and purification.

The heavy chain variable domains of selected antibodies were cloned into a modified pVRC8400 expression vector to produce a full-length human IgG1 heavy chain. IgGs were produced by transient transfection of 293F cells as specified above. At 5 days posttransfection, supernatants were harvested, clarified by low-speed centrifugation, and incubated overnight with protein A agarose resin (GoldBio). The resin was collected in a chromatography column, washed with a column volume of buffer A, and eluted in 0.1 M glycine (pH 2.5), which was immediately neutralized by 1 M Tris (pH 8). Antibodies were then dialyzed against phosphate-buffered saline (PBS) at pH 7.4.

### Recombinant HA expression and purification.

Recombinant HA (rHA) constructs were expressed by infection of insect cells with recombinant baculovirus as previously described (1, 9, 24). In brief, a synthetic DNA corresponding to the full-length ectodomain (FLsE) or the globular HA head was subcloned into a pFastBac vector modified to encode a C-terminal thrombin cleavage site, a T4 fibritin (foldon) trimerization tag, and a 6×His tag. The resulting baculoviruses produce rHA trimers and trimeric HA heads. Monomeric HA heads were produced by subcloning DNAs corresponding to the HA head domain into a pFastBac vector modified to encode a C-terminal rhinovirus 3C protease site and a 6×His tag. The supernatant from recombinant baculovirus-infected High Five cells (*Trichoplusia ni*) was harvested at 72 h postinfection and clarified by centrifugation. Proteins were purified by adsorption to Co-NTA agarose resin, followed by a wash in buffer A, a second wash (trimers only) with buffer A plus 5 to 7 mM imidazole, elution in buffer A plus 350 mM imidazole (pH 8), and gel filtration chromatography on a Superdex 200 column (GE Healthcare) in buffer A.

gHA^shield^ (8) was produced by PEI-facilitated transient transfection of 293F cells maintained in FreeStyle 293 expression medium. Transfection complexes were prepared in Opti-MEM and added to cells. Supernatants were harvested at 4 to 5 days posttransfection and clarified by low-speed centrifugation. Fabs were purified by passage over Co-NTA agarose (Clontech) followed by gel filtration chromatography on a Superdex 200 column (GE Healthcare) in a solution containing 10 mM Tris-HCl and 150 mM NaCl at pH 7.5 (buffer A).

### Biolayer interferometry.

The binding of Fabs with HA heads was analyzed by biolayer interferometry (BLI) (BLItz; forteBIO, Pall); all measurements were performed in buffer A at room temperature. For competition assays, purified His-tagged Fabs were immobilized on a Ni-NTA biosensor. HA head domains and Fab fragments with the His tag removed were used as analytes.

### ELISA.

Five hundred nanograms of rHA FLsE was adhered to high-capacity-binding 96-well plates (Corning) overnight in PBS (pH 7.4) at 4°C. Plates were blocked with PBS containing 2% bovine serum albumin (BSA) and 0.05% Tween 20 (PBS-T) for 1 h at room temperature. Blocking solution was removed, plates were washed once with PBS-T, and 5-fold dilutions of IgGs (in PBS-T) were added to wells. Plates were then incubated for 1 h at room temperature followed by the removal of the IgG solution and three washes with PBS-T. Secondary anti-human IgG-horseradish peroxidase (HRP) (catalog number ab9722; Abcam) diluted 1:20,000 in PBS-T was added to wells and incubated for 30 min at 37°C. Plates were then washed three times with PBS-T. Plates were developed using the one-step ABTS [2,2′-azinobis(3-ethylbenzthiazolinesulfonic acid)] substrate (product number 37615; Thermo Fisher). Following a brief incubation at room temperature, HRP reactions were stopped by the addition of an equal volume of a 1% sodium dodecyl sulfate (SDS) solution. Plates were read on a BioTek ELx808 microplate reader at 405 nm.

Equilibrium dissociation constant (*K_D_*) values for ELISAs were obtained as follows. All dilutions were done in triplicate. The average background signal (no primary antibody) was subtracted from all absorbance values. Values from multiple plates were normalized to the FI6v3 standard that was present on each ELISA plate. The averages from the three measurements were then graphed using GraphPad Prism (v5.0). *K_D_* values were determined by applying a nonlinear fit (one-site binding, hyperbola) to these data points.

### Crystallization.

Fab fragments were coconcentrated with HA head domains at a molar ratio of ∼1:1.3 (Fab to HA head) to a final concentration of ∼20 mg/ml. Crystals of Fab-head complexes were grown in hanging drops over reservoir solutions indicated in Fig. S3 in the supplemental material. Crystals were cryoprotected in the well solution for crystals obtained in polyethylene glycol 400 (PEG 400) at concentrations of ≥30%. Other crystals were cryoprotected with glycerol at concentrations of 12 to 25% in cryoprotectant buffers that were 20% more concentrated than the well solution. The cryoprotectant was added directly to the drop, and crystals were harvested and flash cooled in liquid nitrogen.

10.1128/mBio.01144-21.3 FIG S3 Summary of crystallization conditions and molecular replacement models. Download FIG S3, TIF file, 2.2 MB. Copyright © 2021 McCarthy et al. 2021 McCarthy et al. https://creativecommons.org/licenses/by/4.0/ This content is distributed under the terms of the Creative Commons Attribution 4.0 International license.

### Structure determination and refinement.

We recorded diffraction data at the Advanced Photon Source on beamlines 24-ID-E and 24-ID-C. Data were processed and scaled (XSCALE) with XDS (26). Molecular replacement was carried out with PHASER (27), dividing each complex into four search models (Fig. S3). We carried out refinement calculations with PHENIX (28) and model modifications with COOT (29). Refinement of atomic positions and B factors was followed by translation-liberation-screw (TLS) parameterization and, if applicable, placement of water molecules. All built residues were supported by electron density maps and subsequent rounds of refinement. Final coordinates were validated with the MolProbity server (30). Data collection and refinement statistics are shown in Table S1. Figures were made with PyMOL (Schrödinger, New York, NY).

10.1128/mBio.01144-21.4 TABLE S1 Data collection and refinement statistics. Download Table S1, TIF file, 1.8 MB. Copyright © 2021 McCarthy et al. 2021 McCarthy et al. https://creativecommons.org/licenses/by/4.0/ This content is distributed under the terms of the Creative Commons Attribution 4.0 International license.

### Data availability.

Coordinates and diffraction data have been deposited at the PDB under accession numbers 6XPO, 6XPQ, 6XPR, 6XPX, 6XPY, 6XPZ, 6XQ0, 6XQ2, and 6XQ4.
