# Supplementary figures and images for: A Prevalent Focused Human Antibody Response to the Influenza Virus Hemagglutinin Head Interface
Source: mBio. 2021 Jun 1;12(3):e01144-21. doi: 10.1128/mBio.01144-21 (PMC8262862; doi:10.1128/mBio.01144-21)

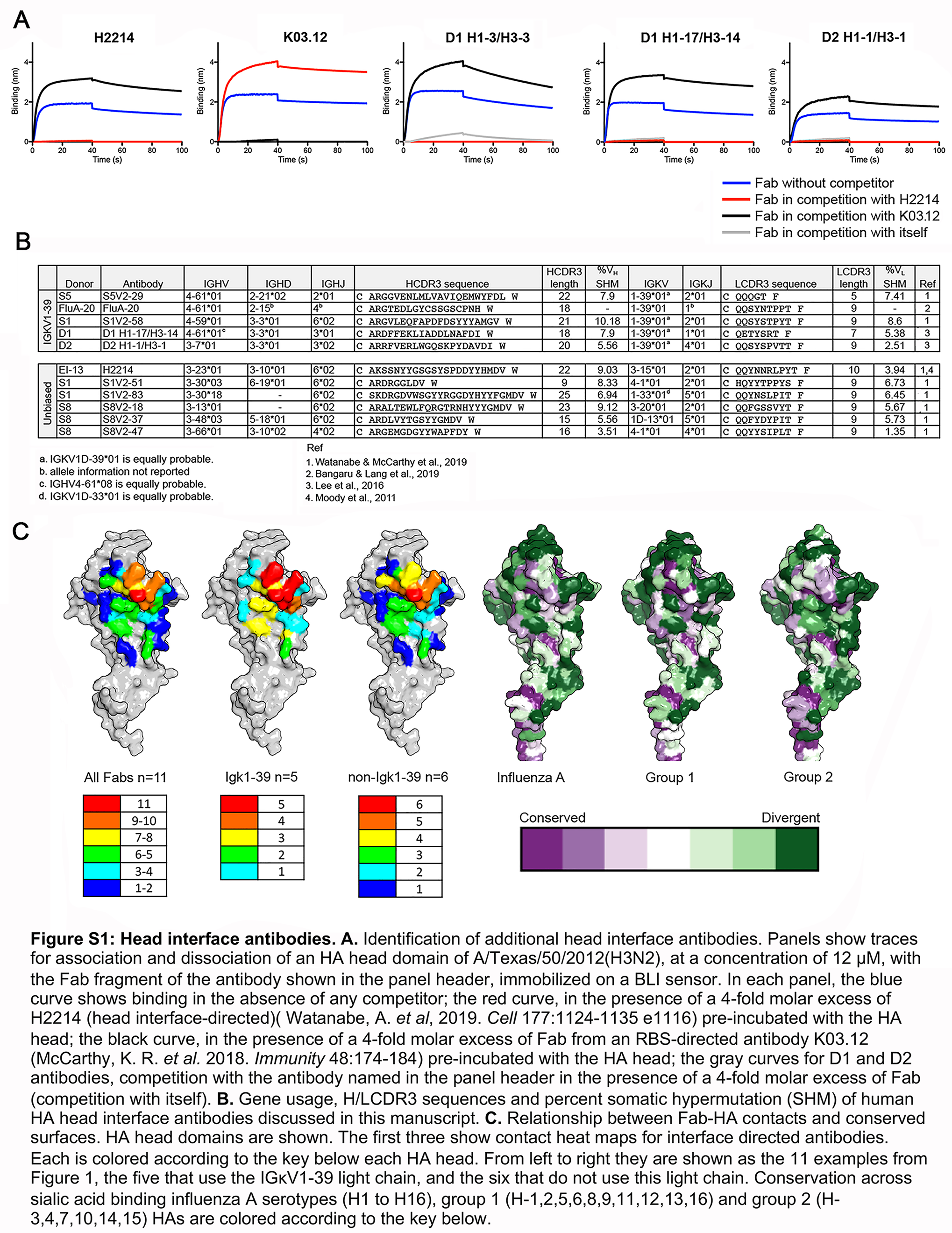

Supplement: FIG S1 [file mbio.01144-21-sf001.tif]

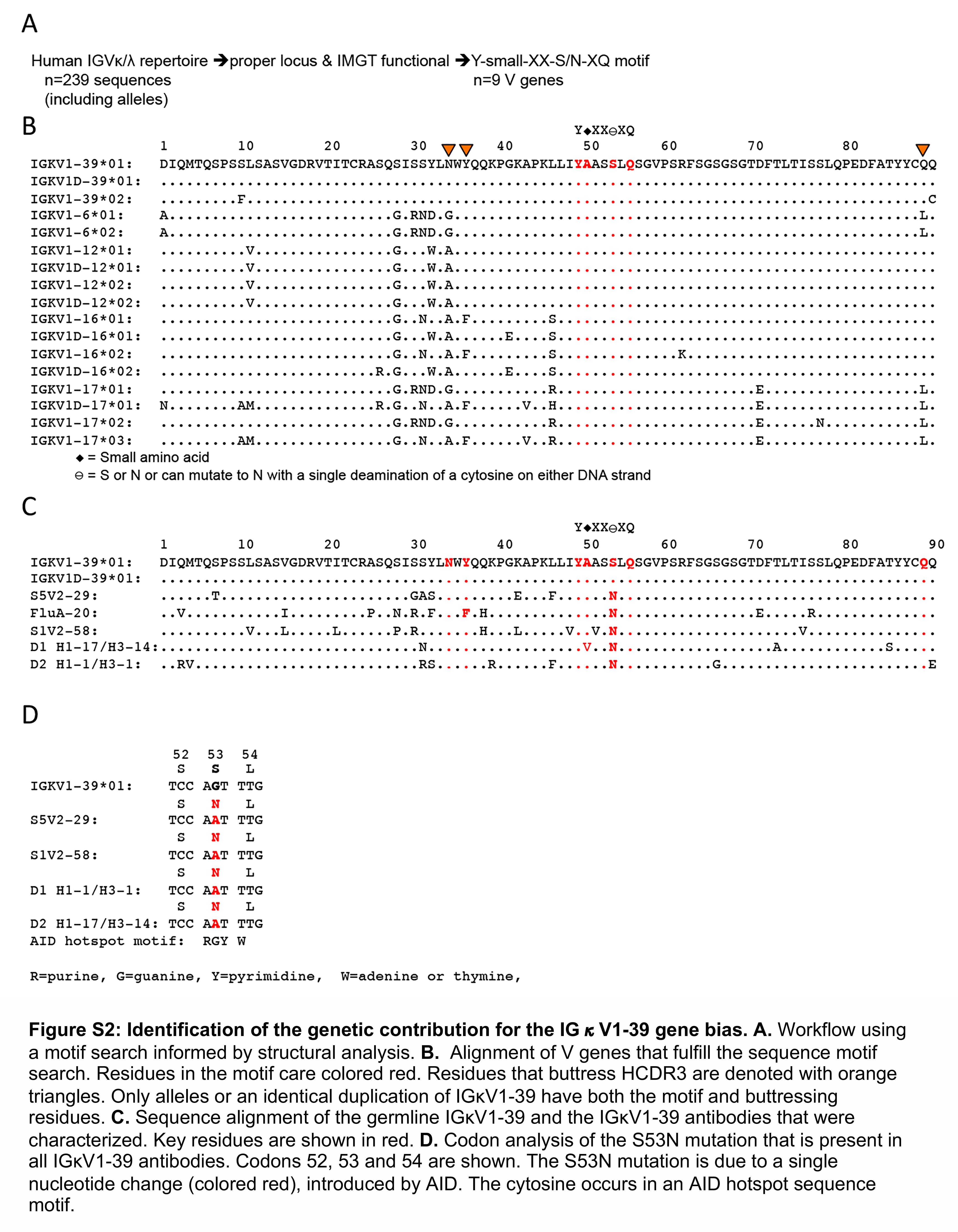

Supplement: FIG S2 [file mbio.01144-21-sf002.tif]

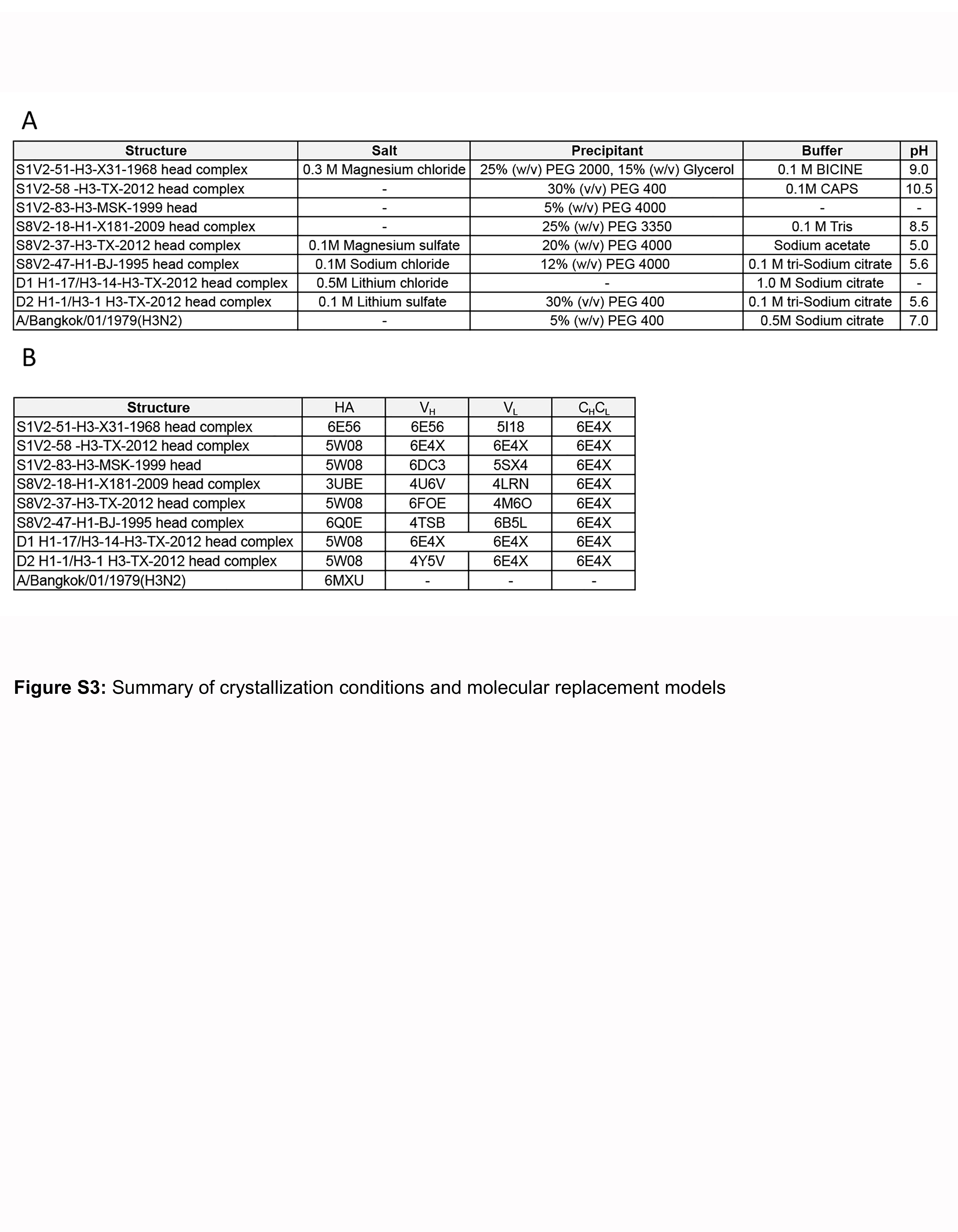

Supplement: FIG S3 [file mbio.01144-21-sf003.tif]

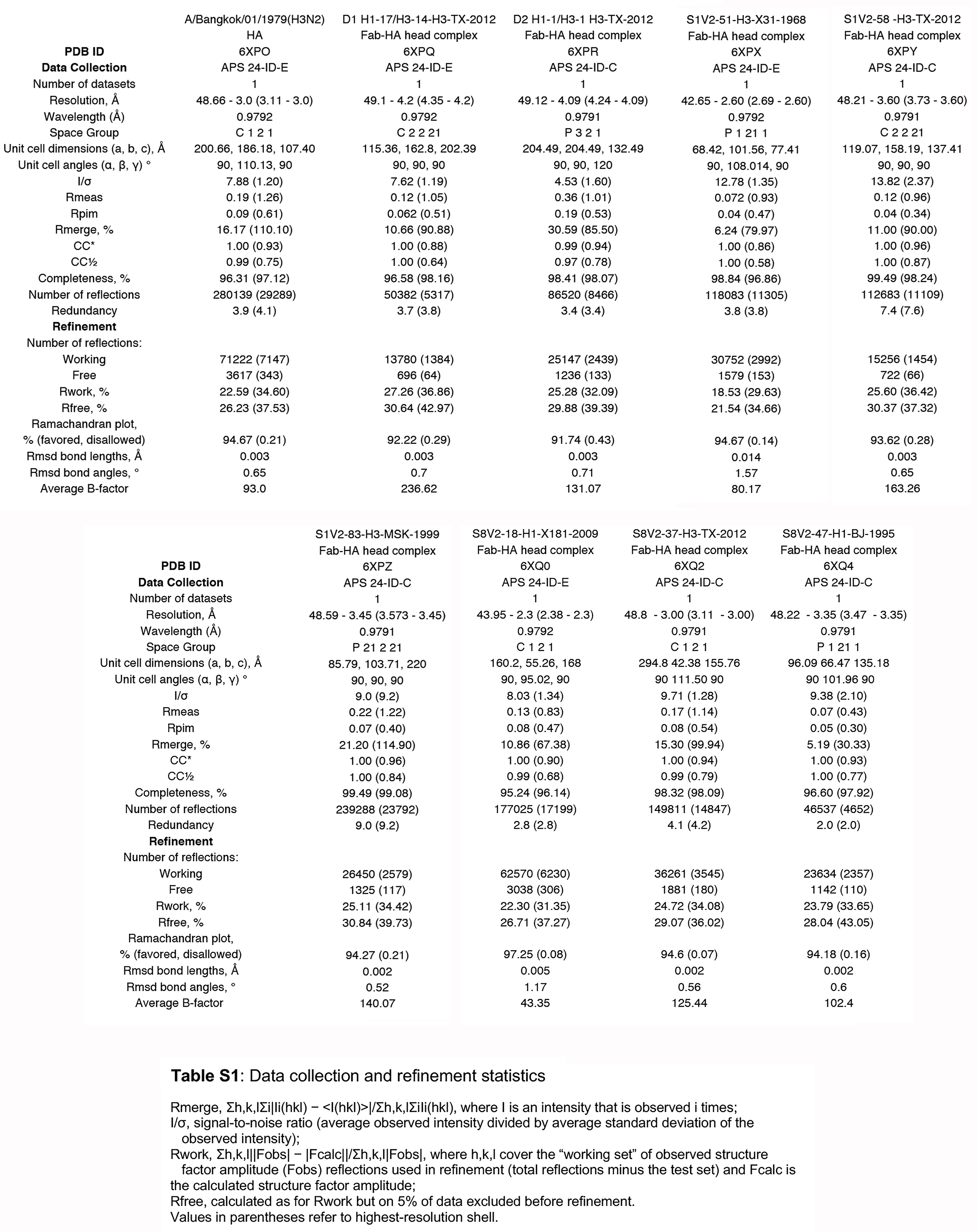

Supplement: TABLE S1 [file mbio.01144-21-st001.tif]
